# Draft Genome Sequence of a Highly Virulent Rabbit *Staphylococcus aureus* Strain

**DOI:** 10.1128/genomeA.00461-15

**Published:** 2015-07-09

**Authors:** Zoltán Német, Ervin Albert, Tibor Nagy, Ferenc Olasz, Endre Barta, János Kiss, Ádám Dán, Krisztián Bányai, Katleen Hermans, Imre Biksi

**Affiliations:** a Faculty of Veterinary Science, Szent István University, Department and Clinic for Production Animals, Üllő, Hungary; b Innovation Centre and Agricultural Biotechnology Institute of the National Agricultural Research, Gödöllő, Hungary; c Veterinary Diagnostic Directorate, National Food Chain Safety Office (NFCSO), Budapest, Hungary; d Institute for Veterinary Medical Research, Hungarian Academy of Sciences, Budapest, Hungary; e Department of Pathology, Bacteriology and Avian Diseases, Ghent University, Ghent, Belgium

## Abstract

We report the draft genome sequence of *Staphylococcus aureus* Sp17, a typical highly virulent (HV) rabbit strain. As current medicine apparently fails to effectively reduce disease and economical losses caused by this organism, it is essential to gain better insight on its genomic arrangement.

## GENOME ANNOUNCEMENT

Staphylococcosis is a disease having major economic impact on industrial rabbit meat production. Infections caused by highly virulent (HV) *Staphylococcus aureus* strains result in severe clinical conditions; these strains are also frequently resistant to antimicrobials. An outbreak of an HV *S. aureus* strain hinders profitable production and frequently necessitates culling the entire flock (1).

A typical HV strain identified as Sp17, originating from a Spanish rabbit farm with severe staphylococcal mastitis problems, was used for whole-genome sequencing (WGS). This strain belongs to the typical highly virulent rabbit *S. aureus* clone, as it shows the mixed biotype CV-C (2), is sensitive to phages of phage group II (3A, 3C, and 71), shows the multiplex PCR pattern specific for highly virulent *S. aureus* strains (3), and has pulsed-field gel electrophoresis type N2 and *spa* type t645 (4).

Total DNA of the strain was subjected to 2 × 300-bp paired-end Illumina MiSeq sequencing at the Department of Biochemistry, Faculty of Medicine, University of Szeged, Hungary. A total of 3.96 million read pairs were recorded, and the estimated coverage of the whole genome is 700×.

The estimated coverage of the subsets of reads was adjusted to 30×, and they were assembled *de novo* using MIRA version 4.0.2 (5), A5 pipeline version 20130326 (6), and SeqMan NGen version 4.1.2 build 25 (DNAStar version 10). Scaffolds were built from different assemblies using Mauve version 2.3.1 (7) as a Geneious version 8.1.2 (8) plugin. This resulted in a total of 10 scaffolds containing 2,684,832 nucleotides. The average G+C content is 32.7%.

Scaffolds were submitted to the RAST annotation server (9). The taxon was set to “*Staphylococcus aureus*” (1280.2034), the genetic code to “11 (*Archaea*, *Bacteria*),” the annotation scheme to “ClassicRAST,” and “preserve gene calls,” “automatically fix errors,” “fix frameshifts,” and “backfill gaps” to “no.” We obtained 2,395 annotated genes, 50 tRNAs, and 5 rRNAs.

A search for similar sequences deposited in GenBank was conducted using BLAST. The best match was *S. aureus* subsp. *aureus* 21337 (accession no. NZ_JHPZ00000000.1). Pairwise alignment showed 98.56% similarity of these sequences, which indicates a close relationship between these strains.

A detailed analysis of the virulence genes in these sequences will be conducted to identify key elements responsible for the remarkable pathogenicity of this strain, in order to facilitate the development of effective treatment methods and/or preventive measures against HV *S. aureus* infections in rabbits.

### Nucleotide sequence accession numbers. 

This whole-genome shotgun project has been deposited at DDBJ/EMBL/GenBank under the accession no. LBCS00000000. The version described in this paper is version LBCS01000000.
